# Enhancement of the antitumor activity of ionising radiation by nimotuzumab, a humanised monoclonal antibody to the epidermal growth factor receptor, in non-small cell lung cancer cell lines of differing epidermal growth factor receptor status

**DOI:** 10.1038/sj.bjc.6604222

**Published:** 2008-02-05

**Authors:** Y Akashi, I Okamoto, T Iwasa, T Yoshida, M Suzuki, E Hatashita, Y Yamada, T Satoh, M Fukuoka, K Ono, K Nakagawa

**Affiliations:** 1 Department of Medical Oncology, Kinki University School of Medicine, 377-2 Ohno-higashi, Osaka-Sayama, Osaka 589-8511, Japan; 2 Radiation Oncology Research Laboratory, Research Reactor Institute, Kyoto University, 2-1010 Asashiro-nishi, Kumatori-cho, Sennan-gun, Osaka 590-0494, Japan

**Keywords:** epidermal growth factor receptor, non-small cell lung cancer, nimotuzumab, monoclonal antibody, genetic alteration, radiosensitisation

## Abstract

The expression and activity of the epidermal growth factor receptor (EGFR) are determinants of radiosensitivity in several tumour types, including non-small cell lung cancer (NSCLC). However, little is known of whether genetic alterations of *EGFR* in NSCLC cells affect the therapeutic response to monoclonal antibodies (mAbs) to EGFR in combination with radiation. We examined the effects of nimotuzumab, a humanised mAb to EGFR, in combination with ionising radiation on human NSCLC cell lines of differing *EGFR* status. Flow cytometry revealed that H292 and Ma-1 cells expressed high and moderate levels of EGFR on the cell surface, respectively, whereas H460, H1299, and H1975 cells showed a low level of surface EGFR expression. Immunoblot analysis revealed that EGFR phosphorylation was inhibited by nimotuzumab in H292 and Ma-1 cells but not in H460, H1299, or H1975 cells. Nimotuzumab augmented the cytotoxic effect of radiation in H292 and Ma-1 cells in a clonogenic assay *in vitro*, with a dose enhancement factor of 1.5 and 1.3, respectively. It also enhanced the antitumor effect of radiation on H292 and Ma-1 cell xenografts in nude mice, with an enhancement factor of 1.3 and 4.0, respectively. Nimotuzumab did not affect the radioresponse of H460 cells *in vitro* or *in vivo*. Nimotuzumab enhanced the antitumor efficacy of radiation in certain human NSCLC cell lines *in vitro* and *in vivo*. This effect may be related to the level of EGFR expression on the cell surface rather than to *EGFR* mutation.

Epidermal growth factor receptor (EGFR) is a receptor tyrosine kinase that is abnormally upregulated and activated in a variety of tumours (Baselga, 2002). Deregulation of receptor tyrosine kinases as a result of overexpression or activating mutations is frequently associated with human cancers and leads to the promotion of cell proliferation or migration, inhibition of cell death, or the induction of angiogenesis (Gschwind *et al*, 2004). The epidermal growth factor receptor has thus been identified as an important target in cancer therapy (Baselga and Arteaga, 2005). Several agents, including small-molecule inhibitors of the tyrosine kinase activity of EGFR (EGFR-TKIs) and monoclonal antibodies (mAbs) specific for EGFR, have been designed to block EGFR signalling selectively (Ettinger, 2006; Harari and Huang, 2006; Imai and Takaoka, 2006). Among EGFR-TKIs, gefitinib and erlotinib have been extensively evaluated in non-small cell lung cancer (NSCLC), and sensitivity to these drugs has been associated with the presence of somatic mutations in the EGFR kinase domain or with *EGFR* amplification (Lynch *et al*, 2004; Paez *et al*, 2004; Pao *et al*, 2004; Cappuzzo *et al*, 2005; Mitsudomi *et al*, 2005; Takano *et al*, 2005). Various mAbs to EGFR are also undergoing preclinical and clinical trials of their efficacy as anticancer agents. However, biological markers able to predict the response to such antibodies have remained elusive.

The possibility of combining chemotherapy or radiation therapy with anti-EGFR mAb treatment has generated much interest, because the cellular targets for these agents and their mechanisms of action are different (Baumann and Krause, 2004). Studies have thus been undertaken to determine whether inhibition of EGFR signalling improves the response to chemotherapy or radiation therapy. Preclinical studies have shown that the anti-EGFR mAb cetuximab markedly increases the cytotoxic effect of chemotherapy or radiation therapy in various EGFR-expressing tumour cell lines (Huang *et al*, 1999; Milas *et al*, 2000; Buchsbaum *et al*, 2002; Prewett *et al*, 2002; Raben *et al*, 2005; Ettinger, 2006). A phase III clinical trial also showed that the combination of cetuximab with radiation therapy resulted in a significant improvement in local control and survival compared with radiation therapy alone, without an increase in radiation-induced side effects, in patients with locally advanced head and neck cancer (Bonner *et al*, 2006).

Nimotuzumab (also known as h-R3) is a humanised anti-EGFR mAb, which is currently undergoing clinical evaluation. In a preclinical study, nimotuzumab showed marked antiproliferative, proapoptotic, and antiangiogenic effects in tumours that overexpress EGFR (Crombet-Ramos *et al*, 2002). In early clinical trials, nimotuzumab has shown a longer half-life and a greater area under the curve (AUC) in comparison with other anti-EGFR antibodies (Crombet *et al*, 2003). A phase I/II trial showed that nimotuzumab was well tolerated and enhanced the curative potential of radiation in patients with advanced head and neck cancer (Crombet *et al*, 2004). Given that little is known of the antitumor action of nimotuzumab in NSCLC, we investigated the growth-inhibitory effects of this mAb alone and in combination with radiation in NSCLC cell lines with differing patterns of EGFR expression. We also examined whether genetic alterations of *EGFR* affect the antitumor action of combined treatment with nimotuzumab and radiation.

## MATERIALS AND METHODS

### Cell lines and reagents

The human NSCLC cell lines NCI-H292 (H292), NCI-H460 (H460), Ma-1, NCI-H1299 (H1299), and NCI-H1975 (H1975) were obtained as previously described (Okabe *et al*, 2007) and were maintained under a humidified atmosphere of 5% CO_2_ in air at 37.0°C in RPMI 1640 medium (Sigma, St Louis, MO, USA) supplemented with 10% fetal bovine serum and 1% penicillin–streptomycin. Nimotuzumab was provided by Daiichi Sankyo Co Ltd (Tokyo, Japan), and gefitinib was obtained from AstraZeneca (Macclesfield, UK).

### Flow cytometric analysis of surface EGFR expression

Cells (1.0 × 10^6^) were stained for 2 h at 4°C with an R-phycoerythrin-conjugated mAb to EGFR (BD Biosciences, San Jose, CA, USA) or an isotype-matched control mAb (BD Biosciences). The cells were washed three times before measurement of fluorescence with a flow cytometer (FACScalibur; Becton Dickinson, San Jose, CA, USA).

### Immunoblot analysis

Cell lysates were fractionated by SDS-polyacrylamide gel electrophoresis on a 7.5% gel, and the separated proteins were transferred to a nitrocellulose membrane. After blocking of nonspecific sites, the membrane was incubated consecutively with primary and secondary antibodies, and immune complexes were detected with the use of enhanced chemiluminescence reagents, as described previously (Okabe *et al*, 2007). Primary antibodies to phosphorylated EGFR (pY1068) were obtained from Cell Signaling Technology (Beverly, MA, USA), and those to EGFR were from Zymed (South San Francisco, CA, USA). Horseradish peroxidase-conjugated goat secondary antibodies were obtained from Amersham Biosciences (Little Chalfont, UK).

### Clonogenic assay

Exponentially growing cells in 25-cm^2^ flasks were harvested by exposure to trypsin and counted. They were diluted serially to appropriate densities and plated in triplicate in 25-cm^2^ flasks containing 10 ml of medium supplemented with 1% fetal bovine serum in the absence or presence of 700 nM nimotuzumab. After incubation for 24 h, the cells were exposed to various doses of *γ*-radiation with a ^60^Co irradiator at a rate of approximately 0.82 Gy min^−1^ and at room temperature. The cells were then washed with phosphate-buffered saline, cultured in drug-free medium for 10–14 days, fixed with methanol : acetic acid (10 : 1, v/v), and stained with crystal violet. Colonies containing >50 cells were counted. The surviving fraction was calculated as (mean number of colonies)/(number of inoculated cells × plating efficiency). Plating efficiency was defined as the mean number of colonies divided by the number of inoculated cells for control cultures not exposed to nimotuzumab or radiation. The surviving fraction for combined treatment was corrected by that for nimotuzumab treatment alone. The dose enhancement factor was then calculated as the dose (Gy) of radiation that yielded a surviving fraction of 0.5 for vehicle-treated cells divided by that for nimotuzumab-treated cells (after correction for drug toxicity).

### Antitumor activity of nimotuzumab with or without radiation *in vivo*

Animal experiments were performed in accordance with the Recommendations for Handling of Laboratory Animals for Biomedical Research, compiled by the Committee on Safety and Ethical Handling Regulations for Laboratory Animal Experiments, Kyoto University, and they met the requirements of the UKCCCR guidelines (Workman *et al*, 1998). Tumour cells (2 × 10^6^) were injected subcutaneously into the right hind leg of 7-week-old female athymic nude mice. tumour volume was determined from caliper measurement of tumour length (*L*) and width (*W*) according to the formula *LW*^2^/2. Treatment was initiated when tumours in each group achieved an average volume of approximately 170–200 mm^3^. Treatment groups consisted of control, nimotuzumab alone, radiation alone, and the combination of nimotuzumab and radiation, with each group containing seven or eight mice. Nimotuzumab was administered intraperitoneally in a single dose of 1.0 mg per mouse; mice in the control and radiation-alone groups were injected with vehicle (physiological saline). Tumours in the right hind leg of mice were exposed to 10 Gy of *γ*-radiation with a ^60^Co irradiator at a rate of approximately 0.32 Gy min^−1^ beginning 6 h after drug treatment. Growth delay (GD) was calculated as the time required for treated tumours to achieve a fivefold increase in volume minus the corresponding time required for control tumours. The enhancement factor was then determined as (GD_combination_–GD_nimotuzumab_)/(GD_radiation_).

## RESULTS

### Surface EGFR expression in NSCLC cell lines of differing *EGFR* status

**Figure 1:**
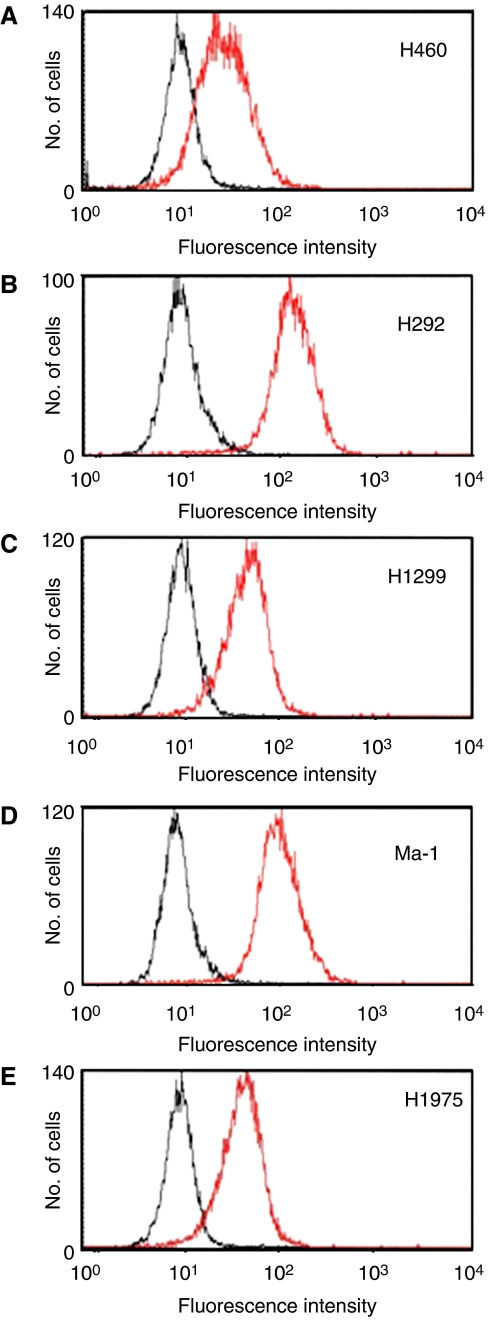
Expression of EGFR on the surface of NSCLC cells. Surface expression of EGFR on H460 (**A**), H292 (**B**), H1299 (**C**), Ma-1 (**D**), and H1975 (**E**) cells was determined by flow cytometry. Representative histograms of cells stained with an anti-EGFR mAb (red peak) or with an isotype-matched control mAb (black peak) are shown.

We first examined the surface expression of EGFR in five NSCLC cell lines by flow cytometry. The *EGFR* status for the cell lines was determined in our previous study (Okabe *et al*, 2007). Three cell lines (H460, H292, and H1299) possess wild-type *EGFR* alleles, whereas the other two cell lines (Ma-1 and H1975) harbour *EGFR* mutations (Table 1). Ma-1 cells have an in-frame deletion in exon 19 (E746–A750). H1975 cells harbour the L858R mutation in exon 21 and a secondary mutation in exon 20 (T790M). Activating mutations in exons 19 and 21 are associated with sensitivity to EGFR-TKIs (Lynch *et al*, 2004; Paez *et al*, 2004; Pao *et al*, 2004; Cappuzzo *et al*, 2005; Mitsudomi *et al*, 2005; Takano *et al*, 2005), whereas the T790M mutation contributes to the development of resistance to these drugs (Kobayashi *et al*, 2005; Pao *et al*, 2005). Our flow cytometric analysis demonstrated that H292 and Ma-1 cells express high and moderate levels of EGFR on the cell surface, respectively, whereas H460, H1299, and H1975 cells showed a low level of surface EGFR expression (Figure 1).

### Effect of nimotuzumab on EGFR phosphorylation

**Figure 2:**
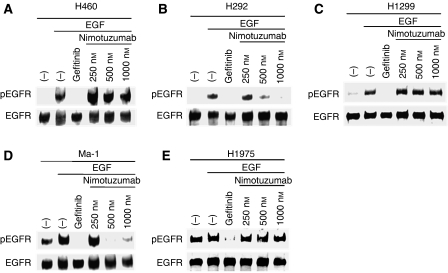
Effect of nimotuzumab on EGFR phosphorylation in NSCLC cells. H460 (**A**), H292 (**B**), H1299 (**C**), Ma-1 (**D**), and H1975 (**E**) cells were deprived of serum overnight and then incubated first for 15 min in the absence or presence of the indicated concentrations of nimotuzumab or gefitinib (10 *μ*M) and then for an additional 15 min in the additional absence or presence of EGF (100 ng ml^−1^). Cell lysates were then subjected to immunoblot analysis with antibodies to the Tyr1068-phosphorylated form of EGFR (pEGFR) as well as with those to total EGFR.

Next, we determined whether nimotuzumab inhibits ligand-induced EGFR phosphorylation in the five NSCLC cell lines. The cells were deprived of serum overnight, exposed to various concentrations of nimotuzumab, or to gefitinib, for 15 min, and then stimulated with EGF for 15 min. In the NSCLC cells that harbour wild-type *EGFR* (H460, H292, and H1299), phosphorylation of EGFR was undetectable in the absence of EGF, but was markedly induced on exposure of the cells to this growth factor. The EGF-induced phosphorylation of EGFR in these cells was completely inhibited by the EGFR-TKI gefitinib. Nimotuzumab also inhibited the EGF-induced EGFR phosphorylation in a concentration-dependent manner in H292 cells (which have a high level of surface EGFR expression), whereas it did not substantially affect such phosphorylation in H460 or H1299 cells (both of which have a low level of surface EGFR expression) (Figure 2A–C). We previously showed that the basal level of EGFR phosphorylation was increased in the *EGFR* mutant NSCLC cell lines Ma-1 and H1975, indicative of constitutive activation of the EGFR tyrosine kinase (Okabe *et al*, 2007). The phosphorylation of EGFR in EGF-treated Ma-1 cells (which have a moderate level of surface EGFR expression) was inhibited by gefitinib as well as by nimotuzumab in a concentration-dependent manner (Figure 2D). In contrast, the constitutive activation of EGFR in H1975 cells (which have a low level of surface EGFR expression) was inhibited partially by gefitinib but was unaffected by nimotuzumab (Figure 2E). These results suggested that the inhibition of EGFR phosphorylation by nimotuzumab may be related to the surface expression level of EGFR rather than to the mutational status of *EGFR*.

### Augmentation of the cytotoxic effect of radiation in NSCLC cells by nimotuzumab *in vitro*

**Figure 3:**
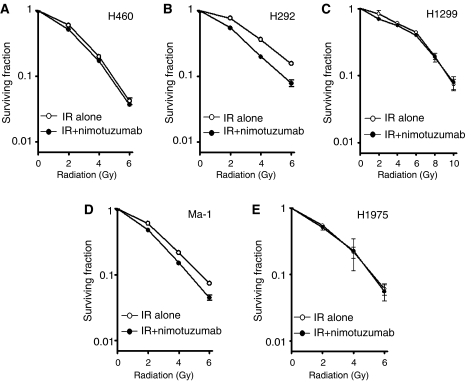
Effect of nimotuzumab on the response of NSCLC cells to radiation *in vitro*. H460 (**A**), H292 (**B**), H1299 (**C**), Ma-1 (**D**), and H1975 (**E**) cells were incubated with or without 700 nM nimotuzumab in medium supplemented with 1% fetal bovine serum for 24 h, exposed to the indicated doses of *γ*-radiation, and then incubated in drug-free medium supplemented with 10% serum for 10–14 days for determination of colony-forming ability. Survival curves were generated after correction of colony formation observed for combined treatment with ionising radiation (IR) and nimotuzumab by that apparent for treatment with nimotuzumab alone. Data are means±s.d. of triplicates from a representative experiment.

We examined whether nimotuzumab might enhance the anticancer effect of *γ*-radiation in the five NSCLC cell lines with the use of a clonogenic assay. Tumour cells were incubated with or without nimotuzumab for 24 h, exposed to various doses of *γ*-radiation, and then allowed to form colonies in drug-free medium for 10–14 days. Survival curves revealed that, whereas nimotuzumab had no effect on the radiation sensitivity of H460, H1299, or H1975 cells, it enhanced the cytotoxic effect of radiation in H292 and Ma-1 cells, with a dose enhancement factor of 1.5 and 1.3, respectively (Figure 3). These results showed that nimotuzumab increased the radiosensitivity of the NSCLC cell lines with high or moderate levels of surface EGFR expression, consistent with the inhibitory effects of this antibody on EGFR signalling.

### Augmentation of the antitumor effect of radiation in NSCLC cells by nimotuzumab *in vivo*

**Figure 4:**
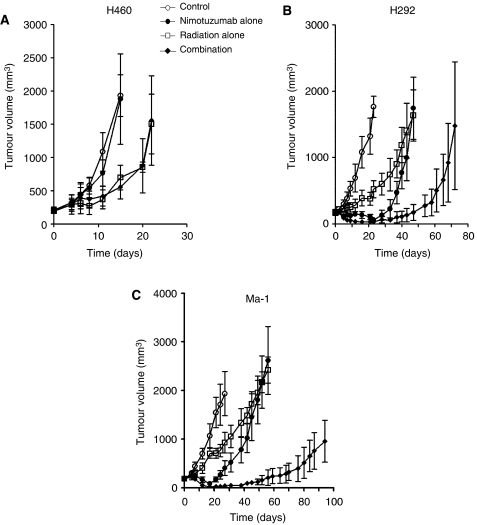
Effect of nimotuzumab on the response of NSCLC cells to radiation *in vivo*. H460 (**A**), H292 (**B**), or Ma-1 (**C**) cells were injected subcutaneously in athymic nude mice. Treatment was initiated when tumours in each group achieved an average volume of approximately 170–200 mm^3^. Mice were treated with a single dose of nimotuzumab (1.0 mg per mouse) intraperitoneally, a single dose of *γ*-radiation (10 Gy), or neither (control) or both modalities, and tumour volume was determined at the indicated time points thereafter. Data are means±s.d. for seven to eight mice per group.

To determine whether the nimotuzumab-induced potentiation of the response of NSCLC cells to radiation observed *in vitro* might also be apparent *in vivo*, we injected three of the cell lines into nude mice to elicit the formation of solid tumours. The mice were then treated with nimotuzumab, radiation, or both modalities. In the H460 xenograft model, tumour growth was inhibited by radiation alone but not by nimotuzumab alone, and the effect of radiation was not promoted by nimotuzumab (Figure 4A). In contrast, radiation and nimotuzumab each inhibited the growth of tumours formed by H292 (Figure 4B) or Ma-1 (Figure 4C) cells during the first few weeks after treatment. Thereafter, the rate of tumour growth increased to a value similar to that seen in control animals. Combined treatment with radiation and nimotuzumab resulted in a substantial delay in tumour growth and subsequent inhibition of the growth rate of H292 and Ma-1 xenografts. The growth delay after treatment with nimotuzumab alone, radiation alone, or both nimotuzumab and radiation was thus 27.2, 19.6, and 53.6 days, respectively, for H292 cells and 26.7, 13.0, and 78.3 days, respectively, for Ma-1 cells (Table 2). The enhancement factor for the effect of nimotuzumab on the efficacy of radiation was 1.3 for H292 cells and 4.0 for Ma-1 cells, revealing the effect to be more than additive. No pronounced tissue damage or toxicities such as diarrhoea or a decrease in body weight of >10% were observed in mice in any of the four treatment groups. These results thus suggested that nimotuzumab potentiated the antitumor activity of radiation in H292 and Ma-1 cells *in vivo* as well as *in vitro*.

## DISCUSSION

Somatic mutations in the EGFR kinase domain and *EGFR* amplification have been associated with a better response to EGFR-TKIs, such as gefitinib and erlotinib, in patients with NSCLC (Lynch *et al*, 2004; Paez *et al*, 2004; Pao *et al*, 2004; Cappuzzo *et al*, 2005; Mitsudomi *et al*, 2005; Takano *et al*, 2005). Given that little is known of the relation between such *EGFR* alterations and the response to treatment with anti-EGFR mAbs, we investigated the antitumor effect of combined treatment with the anti-EGFR mAb nimotuzumab and radiation in NSCLC cell lines of differing *EGFR* status.

The antitumor effect of EGFR-specific mAbs has been thought to result from inhibition of ligand binding to EGFR and consequent inhibition of EGFR activation (Li *et al*, 2005; Marshall, 2006). We, therefore, examined the effect of nimotuzumab on EGF-dependent EGFR signalling. Nimotuzumab inhibited the EGF-induced or constitutive phosphorylation of EGFR in H292 and Ma-1 cells (with high and moderate levels of surface EGFR expression, respectively), consistent with the mode of action of this antibody. However, nimotuzumab did not block EGF-induced or constitutive EGFR phosphorylation in H460, H1299, or H1975 cells (all with a low level of surface EGFR expression). These observations suggest that the inhibitory effect of nimotuzumab on EGFR signalling depends on the expression level of EGFR on the cell surface. A clonogenic cell survival assay revealed that nimotuzumab enhanced the cytotoxic effect of radiation in H292 and Ma-1 cells, but not that in H460, H1299, or H1975 cells. These findings support the notion that the inhibition of EGFR signalling by nimotuzumab is responsible, at least in part, for the enhancement of the cytotoxic effect of radiation by this antibody. Irradiation of tumour cells has been shown to activate EGFR via ligand-independent and ligand-dependent mechanisms, possibly accounting for radiation-induced acceleration of tumour cell repopulation and the development of radioresistance (Schmidt-Ullrich *et al*, 1997, 2003; Dent *et al*, 2003). Such radiation-induced activation of EGFR-dependent processes may represent a rationale for combined treatment with radiation and EGFR inhibitors. It remains to be determined whether nimotuzumab is able to block radiation-induced activation of EGFR.

Consistent with our *in vitro* results, we found that nimotuzumab enhanced the antitumor effect of radiation on H292 or Ma-1 cells in nude mice. Such enhancement was not apparent for tumours formed by H460 cells. Nimotuzumab alone also manifested a substantial antitumor effect for xenografts formed by H292 or Ma-1 cells but not for those formed by H460 cells. Together these results suggest that the efficacy of nimotuzumab monotherapy is a prerequisite for augmentation of radioresponse by this mAb. Nimotuzumab was previously shown to induce the regression of A431 tumour xenografts *in vivo* as a result of inhibition of both tumour cell proliferation and tumour angiogenesis (Crombet-Ramos *et al*, 2002). Immunohistochemical analysis of tumour specimens from head and neck cancer patients treated with the combination of nimotuzumab and radiation also showed evidence of antiproliferative and antiangiogenic effects (Crombet *et al*, 2004). These observations suggest that effects of nimotuzumab on both NSCLC cell proliferation and tumour angiogenesis might contribute to the enhancement of the antitumor efficacy of radiation by this antibody observed in the present study. Enhancement of the anticancer effect of radiation by the anti-EGFR mAb cetuximab was previously shown to be increased by transfection of cells to upregulate the level of EGFR expression, suggesting that potentiation of the antitumor efficacy of radiation by anti-EGFR mAbs is related to the absolute level of EGFR expression (Liang *et al*, 2003; Bonner *et al*, 2004). This finding is consistent with our present results showing that potentiation of the antitumor activity of radiation by nimotuzumab was related to the level of surface EGFR expression. The nimotuzumab-resistant cell line H460 harbours a mutant form of *KRAS* (Balko *et al*, 2006) that has been associated with resistance to cetuximab (Lievre *et al*, 2006). However, we found that nimotuzumab also failed to inhibit EGF-induced EGFR phosphorylation and to enhance the cytotoxic effect of radiation in H1299 cells, which harbour wild-type *KRAS* (Coldren *et al*, 2006). These observations thus support the notion that a low level of EGFR expression at the cell surface is related to resistance to combined treatment with nimotuzumab and radiation, irrespective of *KRAS* status.

We demonstrated that nimotuzumab inhibited EGFR phosphorylation and enhanced the antitumor effect of radiation in *EGFR* mutant Ma-1 cells (with a moderate level of surface EGFR expression) but not in *EGFR*-mutant H1975 cells (with a low level of surface EGFR expression). Nimotuzumab also potentiated the cytotoxic effect of radiation in H292 cells, which harbour wild-type *EGFR* alleles and have a high level of surface EGFR expression. These findings support the notion that *EGFR* mutation is not the major determining factor for enhancement of the antitumor effect of radiation by nimotuzumab, consistent with previous observations with cetuximab (Barber *et al*, 2004; Tsuchihashi *et al*, 2005). However, the mechanisms underlying such enhancement of the antitumor effect of radiation may differ between NSCLC cells harbouring wild-type or mutant *EGFR* alleles. We and others have previously shown that mutations in the tyrosine kinase domain of EGFR are associated with increased ligand-independent tyrosine kinase activity of EGFR (Lynch *et al*, 2004) and aberrant EGFR signalling (Amann *et al*, 2005; Okabe *et al*, 2007). Given that cell-cycle checkpoints activated by ionising radiation are defective in *EGFR*-mutant NSCLC cell lines (Das *et al*, 2006), the constitutive activity of EGFR in such cells may result in unchecked DNA synthesis and in apoptosis on exposure to ionising radiation. It is possible that these defects in *EGFR*-mutant cells affect the enhancement of the antitumor efficacy of radiation by nimotuzumab.

In summary, we have shown that nimotuzumab enhanced the antitumor efficacy of radiation *in vitro* and *in vivo*, providing a rationale for future clinical investigations of the therapeutic efficacy of nimotuzumab in combination with radiotherapy. Our data suggest that potentiation of the antitumor activity of radiation by nimotuzumab may be related to the level of EGFR expression at the cell surface rather than to *EGFR* mutation. The preselection of patients on the basis of genetic factors that predict treatment sensitivity or resistance may thus be required for the combination therapy with nimotuzumab and radiation.

## Figures and Tables

**Table 1:** Characteristics of NSCLC cell lines

**Cell line**	**EGFR surface expression**	***EGFR* status**
H460	Low	Wild type
H292	High	Wild type
H1299	Low	Wild type
Ma-1	Moderate	del(E746–A750)
H1975	Low	L858R/T790M

EGFR=epidermal growth factor receptor; NSCLC=non-small cell lung cancer

**Table 2:** Tumour growth delay in nude mice treated with nimotuzumab, radiation, or both modalities

	**H460**		**H292**		**Ma-1**	
**Treatment**	**Days^a^**	**GD^b^**	**Days**	**GD**	**Days**	**GD**
Control	10.4		13.2		15.1	
Nimotuzumab alone	11.8	1.4	40.4	27.2	41.8	26.7
Radiation alone	20.4	10.0	32.8	19.6	28.1	13.0
Nimotuzumab+radiation	20.5	10.1	66.8	53.6	93.4	78.3
Enhancement factor	0.86		1.3		4.0	

GD=growth delay

a Time required for xenografts in each group to achieve a fivefold increase in volume.

b The additional time (days) required for xenografts in each treatment group to achieve a fivefold increase in volume relative to the corresponding time for xenografts in the control group.
